# Supplementary material for: End of Green Sahara amplified mid- to late Holocene megadroughts in mainland Southeast Asia
Source: Nat Commun. 2020 Aug 21;11:4204. doi: 10.1038/s41467-020-17927-6 (PMC7442841; doi:10.1038/s41467-020-17927-6)
Supplement: Supplementary file 1 — Supplementary Information [file 41467_2020_17927_MOESM1_ESM.pdf]

Supplementary Information

End of Green Sahara amplified Mid-to Late Holocene megadroughts in mainland Southeast Asia

Griffiths *et al.*

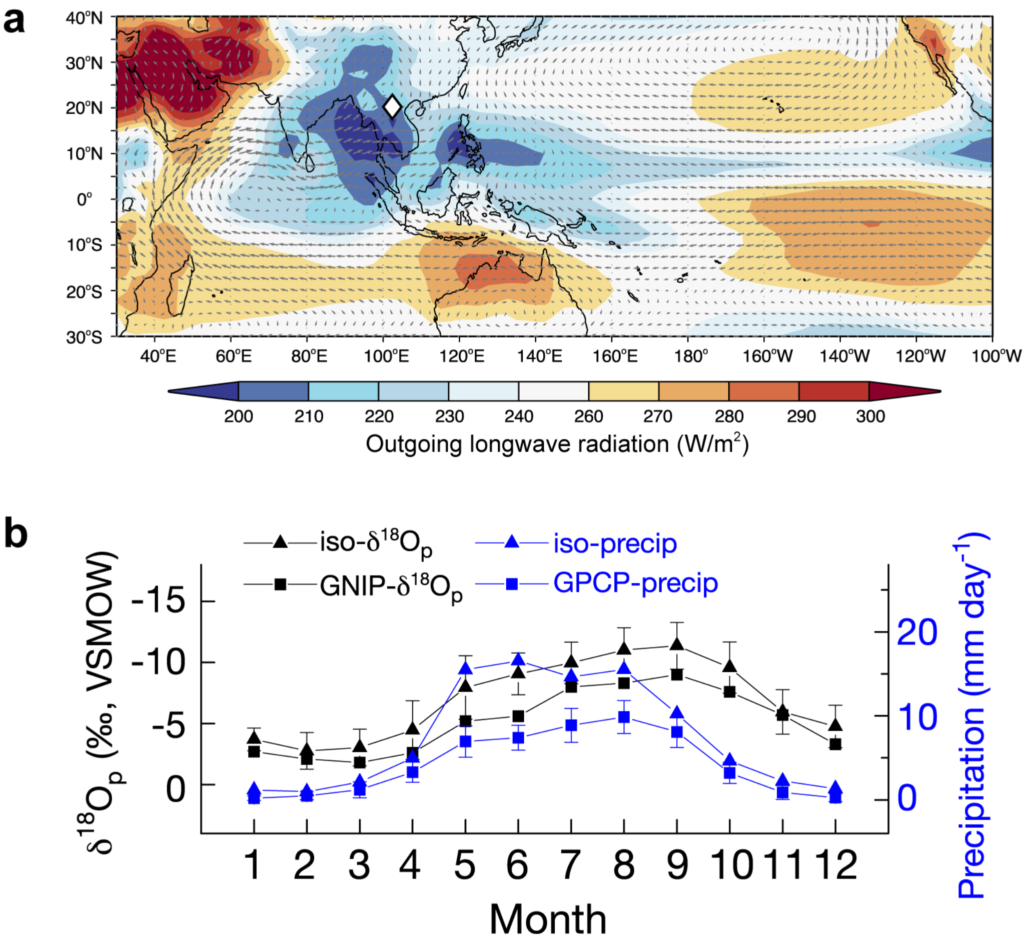

Supplementary Figure 1. Precipitation climatology of mainland Southeast Asia. (a) NCEP-NCAR outgoing longwave radiation (W/m^2; shaded) and JJAS 850 hPa wind vectors (m/s; arrows) averaged over the period 1979-2009. Also indicated is the location of Tham Doun Mai (diamond). (**b)** Average monthly amount-weighted $\delta^{18}O_p$ (black symbols; ‰, VSMOW) and rainfall amount (blue symbols; mm/day) for Tham Doun Mai based on isotope-enabled climate model (IsoGSM) simulations[1] (triangles) and observations (squares). The observed $\delta^{18}O_p$ is based on interpolated GNIP data and observations from the Global Precipitation Climatology Project (GPCP) version 2.2. This figure was adapted from Yang *et al.*[2].

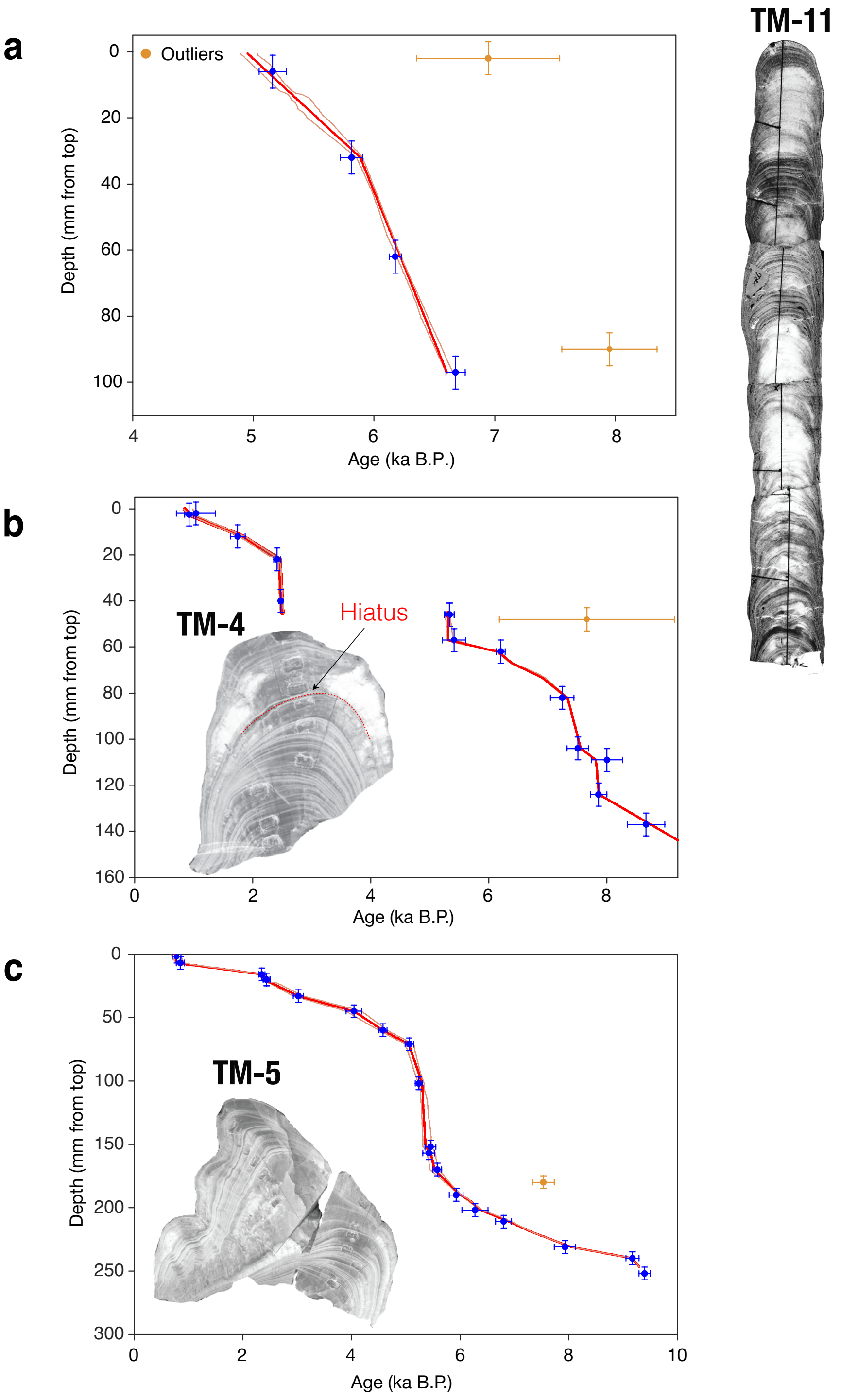

Supplementary Figure 2. Chronologies for Tham Doun Mai speleothems. ^{230}Th-^{234}U ages (blue circles) and age models (red lines) for stalagmites TM11 **(a)**, TM4 **(b)**, and TM5 **(c)**. Age models were derived using Iscam[3], where 2σ uncertainty (pink lines) was calculated using 2,000 pairs of artificially simulated first-order autoregressive time series (AR1) against a red-noise background. Several outliers (orange circles) were omitted from the dataset prior to analysis.

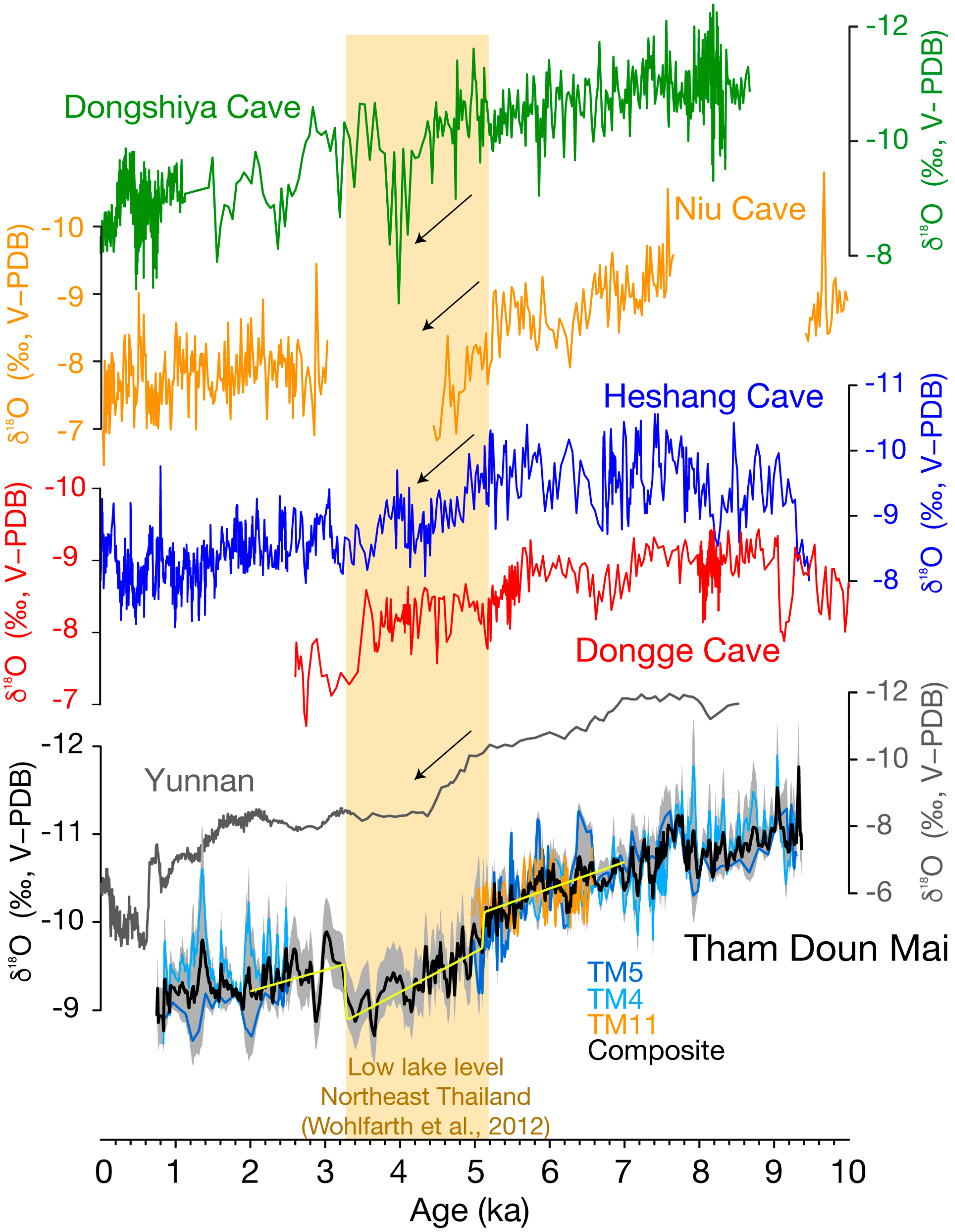

Supplementary Figure 3. Oxygen isotope records from East Asia. $\delta^{18}O$ records from Dongshiya Cave (northern China; green)[4], Niu Cave (central China; orange)[5], Heshang Cave (central China; blue)[6], Dongge Cave (southern China; red)[7], and Xingyun Lake (Yunnan, southern China; gray)[8], all show trends towards more enriched $\delta^{18}O$ values beginning at ≈5 ka, consistent with Tham Doun Mai (this study; black). This period of weakening in the East Asian monsoon is also consistent with drying out of Lake Kumphawapi[9] (shaded vertical bar), the largest natural freshwater lake of northeast Thailand.

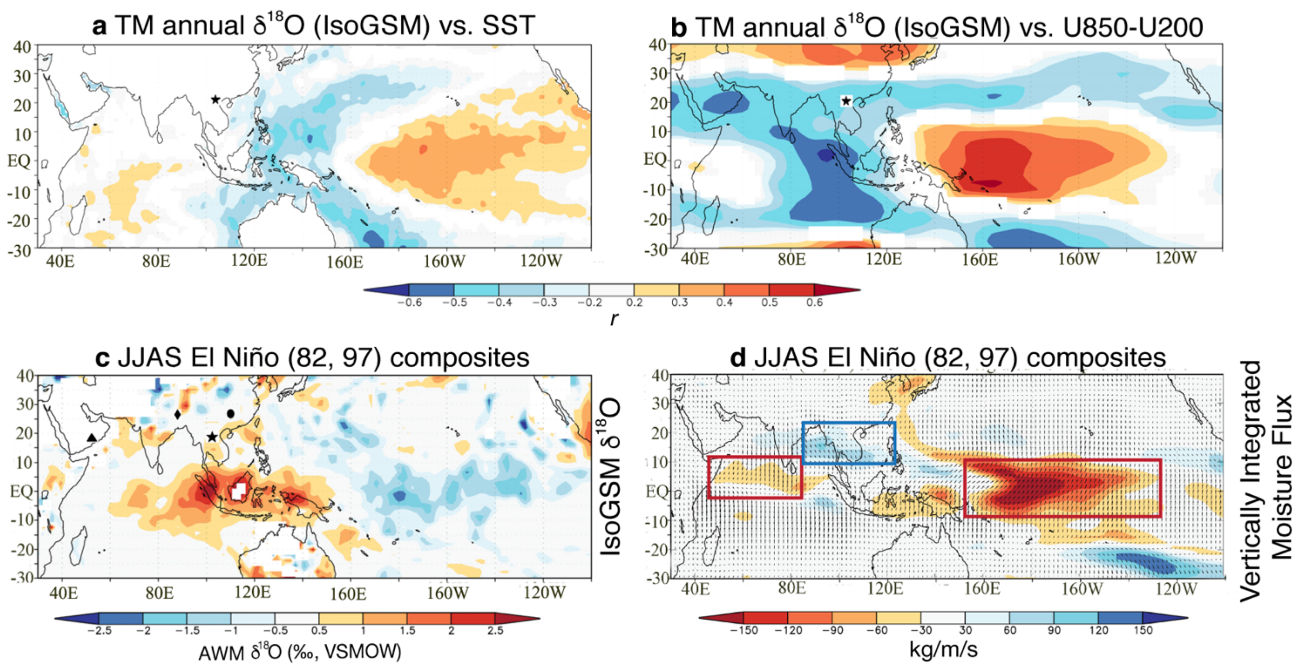

Supplementary Figure 4. Modern $\delta^{18}O_p$ systematics at Tham Doun Mai. Field correlation maps between Tham Doun Mai (star) annual amount-weighted $\delta^{18}O_p$ [based on isotope-enabled climate model (IsoGSM) simulations] versus **(a)** annual sea surface temperatures (HadSST1) and **(b)** annual NOAA vertical wind shear (U_{850}-U_{200}) for the period 1979-2009. Colors represent significant correlation coefficients (*r* values) at the 90% level. **(c)** June-September (JJAS) composites of amount-weighted $\delta^{18}O_p$, and **(d)** vertically integrated moisture flux for the 1982 and 1997 El Niño events. Figure adapted from Yang *et al.*[2].

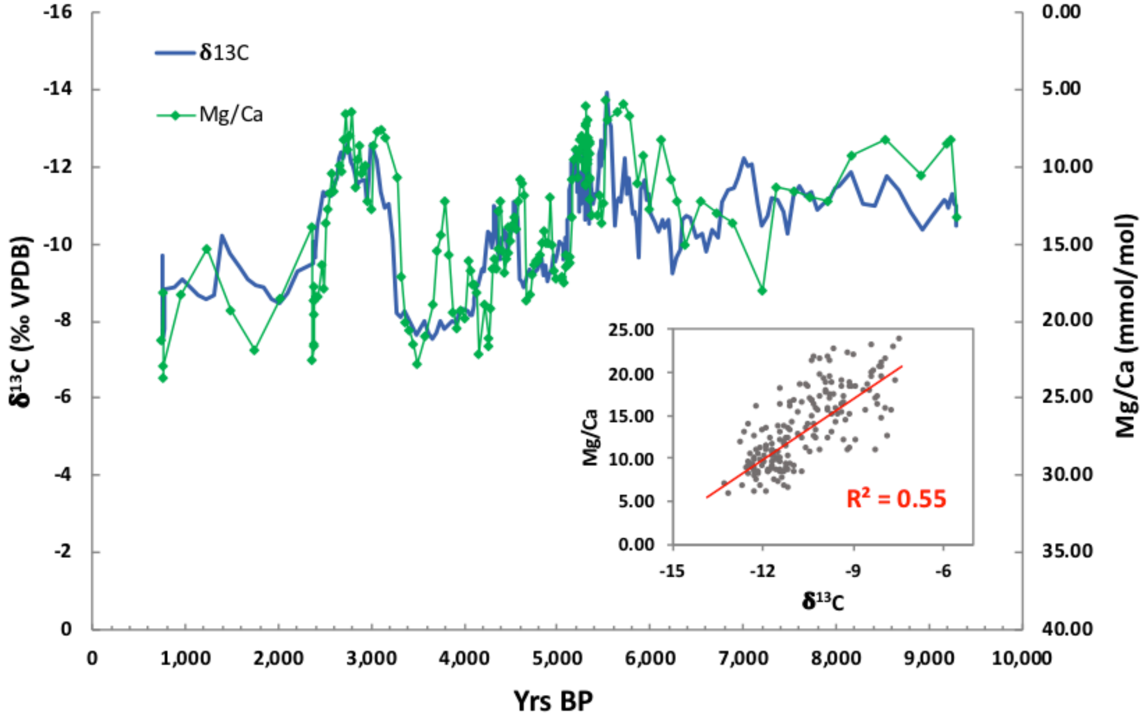

Supplementary Figure 5. Multiproxy evidence for Holocene changes in Tham Doun Mai hydroclimate. Comparison of $\delta^{13}C$ (blue) and Mg/Ca (green) for stalagmite TM5. Inset: Cross plot of $\delta^{13}C$ vs. Mg/Ca.

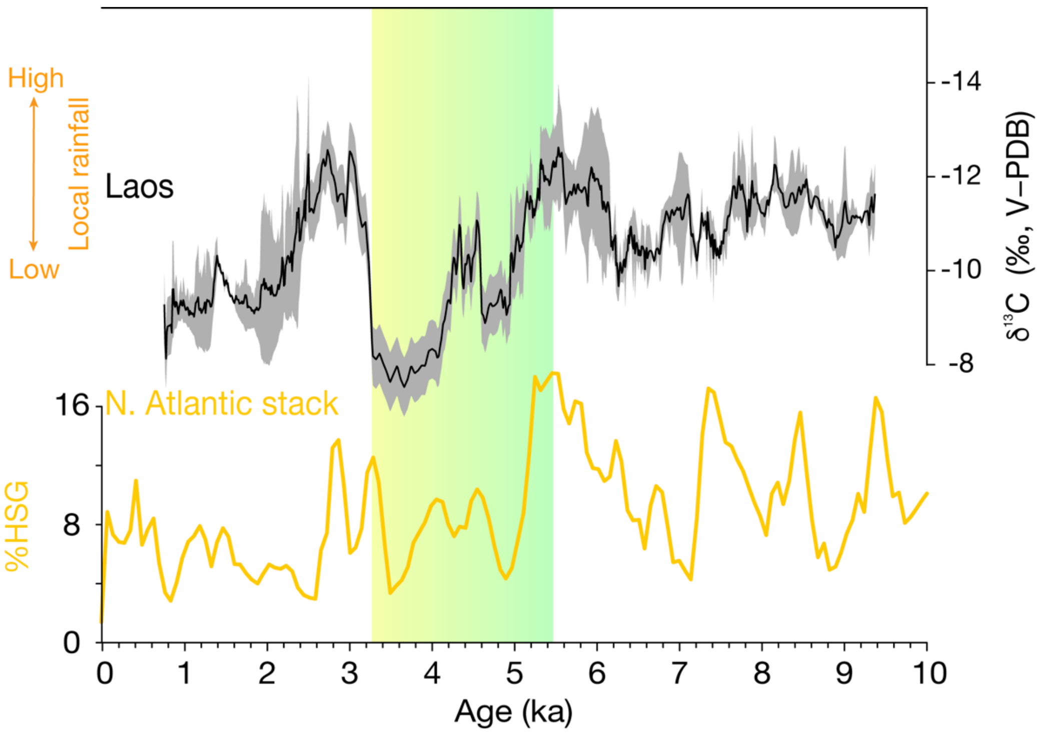

Supplementary Figure 6. Comparison between climate in Southeast Asia and the North Atlantic. Northern Laos composite $\delta^{13}C$ record (black line: average between TM4, TM5, and TM11; gray shading: 1σ uncertainty) from Tham Doun Mai speleothems compared with a stacked hematite-stained grain (HSG) record from three sediment cores located in the North Atlantic (orange line)[10]. Vertical shaded bar indicates the approximate timing of the end-Green Sahara.

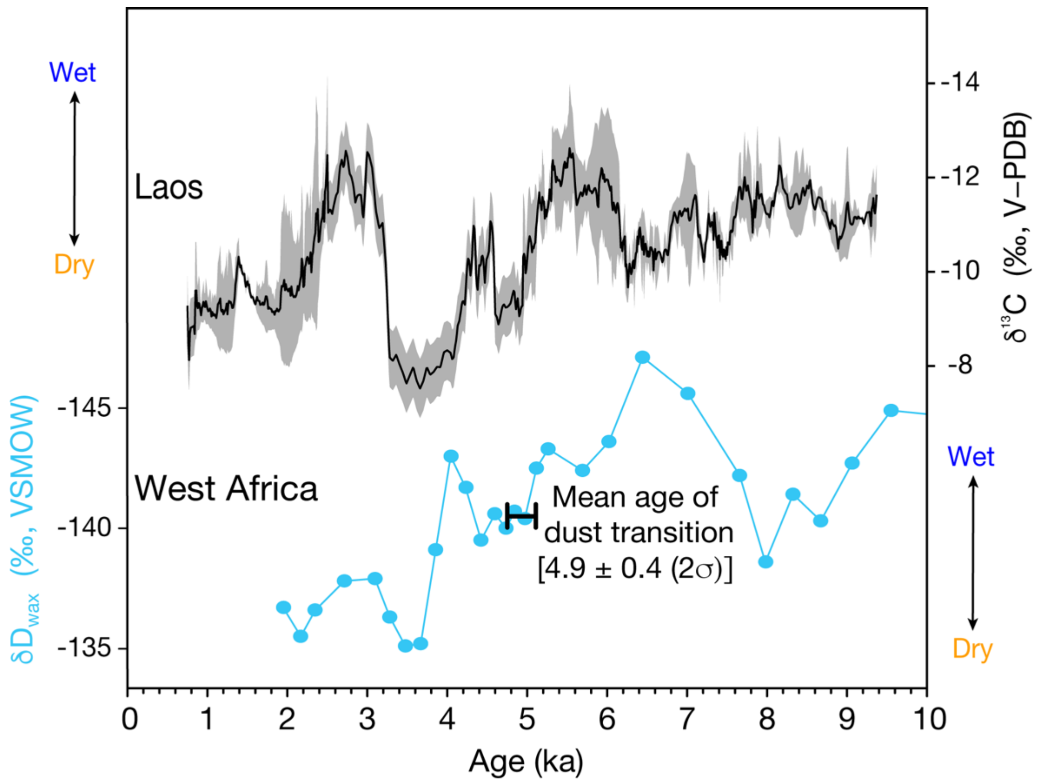

Supplementary Figure 7. Southeast Asian hydroclimate and links with the end of the Green Sahara. The onset of megadrought conditions in Northern Laos at ~5 ka, inferred from the Tham Doun Mai composite $\delta^{13}C$ record, generally coincides with the end of the African Humid Period from the perspective of δD of leaf wax[11] and dust[12] records from West Africa.

Supplementary Table 1. U-Th ages for Tham Doun Mai stalagmites TM4, TM5, and TM11. "Depth from top" is the distance of each sample from the top of the stalagmite. All measured ratios are activity ratios. Ages were calculated using half-lives of Cheng *et al.*[13]. Ages were corrected for small amounts of initial Th utilizing the average crustal (^{230}Th/^{232}Th) value of 1.21±50%. The uncertainties corresponding to this initial Th correction are arbitrarily assigned to be 50% of the (^{230}Th/^{232}Th) ratio. 95% confidence intervals for the ages are calculated using an internally developed Monte Carlo simulation. Initial (^{234}U/^{238}U) is calculated using the corrected age. Grey-shaded samples were treated as outliers and thus were not included in the age-model calculation.

OX ID	UCI ID	Year measured	Depth from top (cm)	^{238}U (ppm)	^{232}Th (ppb)	(^{230}Th/^{232}Th)	2s	(^{230}Th/^{238}U)	2s	(^{234}U/^{238}U) initial (m/c mean):	2s	^{230}Th/^{238}U age (m/c mean), raw age:	2s	^{230}Th/^{238}U age (m/c mean), initial Th corrected:	2s	^{230}Th/^{238}U age (m/c mean), raw age: (Yrs BP)	2s	^{230}Th/^{238}U age (m/c mean), initial Th corrected: (Yrs BP)	2s
TM5																			
TB5_5	TM5-H5	2015	0.2	0.21	0.19	39.5	3.6	0.0116	0.0010	1.4714	0.0064	867	-78	844	82	802	-78	779	82
HY	UTM5A-1	2012	0.7	0.18	0.25	27.8	2.3	0.0127	0.0009	1.4693	0.0059	944	66	908	74	882	66	846	74
TB5_4	TM5-H4	2015	1.6	0.21	0.08	263.5	9.4	0.0317	0.0007	1.4440	0.0063	2420	-57	2411	58	2355	-57	2346	58
HY	UTM5A-2	2012	2	0.21	0.01	1971.6	2006.7	0.0329	0.0009	1.4522	0.0061	2497	66	2496	65	2435	66	2434	65
HY	UTM5A-2	2012	2	0.21	0.01	1971.6	2032.5	0.0329	0.0008	1.4532	0.0070	2496	64	2495	64	2434	64	2433	64
HY	UTM5A-3	2012	3.3	0.23	0.78	39.0	0.7	0.0422	0.0006	1.4773	0.0127	3166	55	3082	95	3104	55	3020	95
HY	UTM5A-4	2012	4.5	0.25	0.85	50.2	1.6	0.0556	0.0014	1.4773	0.0058	4190	112	4104	144	4128	112	4042	144
HY	UTM5A-5	2012	6	0.36	0.61	111.3	2.8	0.0620	0.0008	1.4774	0.0072	4683	63	4639	75	4621	63	4577	75
HY	UTM5A-6	2012	7.1	0.26	0.22	236.1	14.0	0.0671	0.0009	1.4586	0.0059	5146	76	5124	80	5084	76	5062	80
HY	UTM5A-7	2012	10.2	0.35	0.18	400.4	32.2	0.0693	0.0008	1.4609	0.0057	5311	64	5298	65	5249	64	5236	65
HY	UTM5A-8	2012	15.2	0.20	0.37	117.6	4.1	0.0718	0.0011	1.4477	0.0056	5564	86	5516	99	5502	86	5454	99
HY	UTM5B-1	2012	15.7	0.28	0.75	81.4	2.0	0.0722	0.0012	1.4569	0.0053	5554	91	5484	112	5492	91	5422	112
TB5_1	TM5-H1	2015	17	0.18	0.12	329.9	6.4	0.0729	0.0009	1.4455	0.0062	5659	-77	5641	80	5594	-77	5576	80
HY	UTM5B-2	2012	18	0.20	1.47	41.6	0.6	0.0987	0.0011	1.4382	0.0069	7782	94	7592	198	7720	94	7530	198
HY	UTM5B-3	2012	19	0.19	0.09	523.1	112.1	0.0771	0.0015	1.4435	0.0061	6001	126	5989	127	5939	126	5927	127
HY	UTM5B-4	2012	20.2	0.22	2.24	26.0	0.4	0.0854	0.0010	1.4609	0.0053	6591	81	6334	243	6529	81	6272	243
HY	UTM5B-5	2012	21.1	0.17	0.17	268.5	31.5	0.0900	0.0018	1.4742	0.0078	6888	146	6862	147	6826	146	6800	147
TB5_2	TM5-H2	2015	23.1	0.45	3.68	39.9	0.3	0.1067	0.0007	1.4768	0.0062	8204	-56	7994	196	8139	-56	7929	196
HY	UTM5B-6	2012	24	0.15	0.05	1096.7	307.9	0.1155	0.0015	1.4271	0.0079	9241	120	9232	119	9179	120	9170	119
TB5_3	TM5-H3	2015	25.2	0.20	0.15	493.4	7.5	0.1186	0.0012	1.4299	0.0058	9479	-102	9460	103	9414	-102	9395	103
TM4																			
TB6_7	TM4-H5	2015	0.2	0.12	0.22	25.9	10.0	0.0157	0.0046	1.5001	0.0071	1148	-342	1101	331	1083	-342	1036	331
HY	UTM4-1	2012	0.25	0.12	0.05	89.6	17.9	0.0135	0.0010	1.4913	0.0061	993	77	982	77	931	77	920	77
TB6_6	TM4-H4	2015	1.2	0.25	0.63	29.4	2.7	0.0248	0.0015	1.4602	0.0057	1873	-111	1807	126	1808	-111	1742	126
HY	UTM4-2	2012	2.2	0.27	0.00	9753.0	47765.5	0.0326	0.0007	1.4581	0.0050	2472	53	2472	53	2410	53	2410	53
HY	UTM4-3	2012	4	0.23	0.00	6400.8	12433.0	0.0335	0.0005	1.4609	0.0060	2534	37	2534	37	2472	37	2472	37
HY	UTM4-4	2012	4.6	0.20	0.03	1393.1	555.3	0.0695	0.0011	1.4423	0.0058	5394	84	5390	83	5332	84	5328	83
HY	UTM4-4	2012	4.6	0.20	0.03	1393.1	550.2	0.0695	0.0010	1.4423	0.0058	5398	82	5395	81	5336	82	5333	81
TB6_3	TM4-H1	2015	4.8	0.25	16.17	5.6	0.1	0.1196	0.0020	1.4525	0.0061	9397	-166	7723	1485	9332	-166	7658	1485
TB6_4	TM4-H2	2015	5.7	0.23	0.19	261.9	60.1	0.0707	0.0025	1.4413	0.0057	5495	-201	5474	199	5430	-201	5409	199
HY	UTM4-5	2012	6.2	0.22	0.34	164.8	5.6	0.0825	0.0008	1.4737	0.0059	6299	65	6260	73	6237	65	6198	73
HY	UTM4-6	2012	8.2	0.28	2.30	36.2	0.4	0.0984	0.0009	1.4838	0.0061	7511	71	7301	201	7449	71	7239	201
HY	UTM4-7	2012	10.4	0.23	1.43	50.3	1.0	0.1006	0.0014	1.4774	0.0068	7721	110	7567	182	7659	110	7505	182
TB6_5	TM4-H3	2015	10.9	0.21	1.88	38.2	1.2	0.1094	0.0020	1.5020	0.0061	8279	-161	8063	261	8214	-161	7998	261
HY	UTM4-8	2012	12.4	0.22	0.74	92.8	2.2	0.1024	0.0013	1.4513	0.0058	8004	106	7917	138	7942	106	7855	138
HY	UTM4-9	2012	13.7	0.23	2.99	27.8	0.4	0.1163	0.0013	1.4655	0.0075	9049	109	8724	319	8987	109	8662	319

Supplementary Table 1 continued.

TM11																			
LE	TM11D-1	2012	0.2	0.36	10.52	11.8	0.4	0.1129	0.0016	1.6726	0.0081	7651	117	7008	593	7589	117	6946	593
TB5_6	TM11-H1	2015	0.6	0.24	0.86	66.9	0.9	0.0790	0.0010	1.6674	0.0069	5307	-72	5226	111	5242	-72	5161	111
HY	TM11D-2	2012	3.2	0.28	1.00	78.7	1.1	0.0931	0.0009	1.7582	0.0067	5953	59	5876	93	5891	59	5814	93
HY	TM11D-3	2012	6.2	0.35	0.32	309.0	10.2	0.0931	0.0007	1.6753	0.0062	6259	47	6238	50	6197	47	6176	50
LE	TM11D-4	2012	9	0.29	5.64	20.2	0.7	0.1283	0.0018	1.7334	0.0083	8427	127	8013	394	8365	127	7951	394
HY	TM11D-5	2012	9.7	0.28	0.83	106.1	1.5	0.1016	0.0007	1.6866	0.0062	6802	50	6737	79	6740	50	6675	79
TB5_7	TM11-H2	2015	11.9	0.35	1.52	78.7	0.5	0.1124	0.0006	1.6364	0.0069	7793	-49	7694	102	7728	-49	7629	102
HY	TM11C-1	2012	12.7	0.36	1.08	123.0	2.7	0.1196	0.0012	1.6474	0.0063	8256	89	8189	110	8194	89	8127	110
AM33-1	TM11-U1	2014	14.3	0.36	0.55	234.2	14.9	0.1161	0.0012	1.6411	0.0065	8039	85	8005	89	7975	85	7941	89
LE	TM11C-2	2012	15.1	0.35	2.49	61.5	2.1	0.1416	0.0019	1.6519	0.0081	9826	143	9667	205	9764	143	9605	205
HY	TM11C-3	2012	16.5	0.79	2.32	120.6	1.3	0.1150	0.0007	1.5774	0.0059	8291	53	8223	85	8229	53	8161	85
HY	TM11C-4	2012	16.9	0.28	0.59	154.2	2.5	0.1056	0.0007	1.6128	0.0060	7416	49	7369	69	7354	49	7307	69
LE	TM11C-5	2012	19.2	0.35	0.18	600.1	22.1	0.1031	0.0015	1.5146	0.0071	7717	116	7704	116	7655	116	7642	116
HY	TM11B-1	2012	22.9	0.34	0.46	247.3	14.2	0.1104	0.0016	1.6312	0.0064	7673	112	7642	118	7611	112	7580	118
AM33-2	TM11-U2	2014	22.9	0.33	0.31	365.1	40.5	0.1101	0.0015	1.6227	0.0063	7696	103	7674	105	7632	103	7610	105
HY	TM11B-2	2012	25.7	0.32	0.78	155.7	2.8	0.1244	0.0009	1.6700	0.0063	8484	66	8430	87	8422	66	8368	87
TB5_8	TM11-H3	2015	27.5	0.35	0.54	254.7	2.8	0.1282	0.0012	1.6540	0.0069	8846	-87	8811	92	8781	-87	8746	92
LE	TM11A-1	2012	29.3	0.30	0.81	151.5	5.2	0.1329	0.0019	1.7336	0.0081	8747	130	8690	141	8685	130	8628	141
AM33-3	TM11-U3	2014	30.4	0.28	0.23	571.1	92.6	0.1499	0.0015	1.8756	0.0076	9144	98	9128	97	9080	98	9064	97
HY	TM11A-2	2012	32.9	0.34	3.54	47.6	0.4	0.1623	0.0010	1.8141	0.0071	10285	73	10068	205	10223	73	10006	205
AM33-5	TM11-U4	2014	35.3	0.19	3.92	33.9	0.5	0.2304	0.0025	2.0937	0.0085	12835	149	12460	372	12771	149	12396	372
TB5_9	TM11-H4	2015	37	0.39	0.94	186.1	1.7	0.1483	0.0012	1.6356	0.0069	10425	-93	10370	106	10360	-93	10305	106
LE	TM11A-3	2012	37.2	0.39	0.48	351.2	12.1	0.1409	0.0019	1.6440	0.0077	9826	141	9798	141	9764	141	9736	141

Supplementary Table 2. Sites mentioned in Figure 1. Blue (red) text indicates stronger (weaker) monsoon and/or wetter (drier) conditions at those locations at ~4 ka as inferred from the interpretation of the proxies (e.g., lower $\delta^{18}O$ in the speleothem records reflect a strong monsoon) in the original publications.

Site name	Latitude	Longitude	Climate archive
Shennong Cave[14]	28.7	117.3	Speleothem
Xiangshui Cave[15]	25.3	110.9	Speleothem
Xianglong Cave[16]	33.0	106.3	Speleothem
East China Sea (KY07-04-1)[17]	31.6	128.9	Marine sediment
Tengchongqinghai Lake[18]	27.4	98.6	Lake
Lake Changhu[19]	30.5	112.5	Lake
Daiyunshan peat[20]	25.8	118.3	Peat
Dahu swamp[21,22]	24.8	115.0	Peat
Dajiuhu peat[23,24]	31.3	110.0	Peat
Daping peat[25]	26.2	110.1	Peat
SZY peat bog, Gantang Village[26]	26.8	119.0	Peat
Chengjiachuan site, Jinghe River[27]	34.5	108.0	Flood deposits
Huxizhuang loess–soil profile, Qishuihe River[28]	34.3	108.1	Flood deposits
Yellow River, Guanting Basin[19]	35.9	102.8	Flood deposits
Nuanhe Cave[29]	41.3	124.9	Speleothem
Dongge Cave[30]	25.3	108.1	Speleothem
Dark Cave[31]	27.2	106.2	Speleothem
Mawmluh Cave[32]	25.3	91.9	Speleothem
Daihai Lake[33]	40.6	112.7	Lake
Erhai Lake[34]	25.8	100.2	Lake
Tianchi Lake[35]	35.25	106.3	Lake
Gonghai Lake[36]	38.9	112.2	Lake
Dali Lake[37]	43.2	116.5	Lake
Hulun Lake[38]	49.0	117.4	Lake
Taihu Lake[39]	31.1	118.7	Lake
Lake Huguang Maar[40]	21.2	110.3	Lake
Xingyun Lake[8]	24.2	102.8	Lake
Lake Kumphawapi[9]	17.2	103.0	Lake
Hongyuan peat[41]	32.8	102.5	Peat

Supplementary Table 3. Radiocarbon results for stalagmite TM5. The DCP was calculated using the formula: DCP = 1 – ($a^{14}C_{stal.init.}$ / $a^{14}C_{atm.init.}$) where $a^{14}C_{stal.init.}$ and $a^{14}C_{atm.init.}$ represent the speleothem and atmospheric ^{14}C activity, respectively, at the time of calcite precipitation.

Sample ID	U/Th Age	$a^{14}C_{meas}$ [pMC]	$a^{14}C_{stal\ init}$ [pMC]	$a^{14}C_{atm.init.}$ [pMC]	DCP [%]
TDM-MG14C-1	2384±28	63.6±0.1	84.9	98.7	14.0
TDM-MG14C-2	2753±82	64.9±0.1	90.6	100.0	9.4
TDM-MG14C-3	3063±97	63.3±0.1	91.6	101.1	9.4
TDM-MG14C-4	3616±124	60.3±0.1	93.4	101.9	8.4
TDM-MG14C-5	3786±132	59.5±0.1	94.0	102.3	8.1
TDM-MG14C-6	4113±135	56±0.1	92.1	103.1	10.6
TDM-MG14C-7	4256±116	54.9±0.1	91.8	103.8	11.5
TDM-MG14C-8	4309±110	55.3±0.1	93.2	104.3	10.7
TDM-MG14C-9	4577±75	53.1±0.1	92.4	104.5	11.6
TDM-MG14C-10	4642±76	52.2±0.1	91.5	104.7	12.6
TDM-MG14C-11	4923±79	51.1±0.1	92.7	105.1	11.8
TDM-MG14C-12	5101±68	49.8±0.1	92.3	106.0	12.9
TDM-MG14C-13	5152±61	49.6±0.1	92.5	107.3	13.8
TDM-MG14C-14	5204±55	48.9±0.1	91.7	108.1	15.1
TDM-MG14C-15	5283±52	48.8±0.1	92.5	106.4	13.0

Supplementary References

1 Yoshimura, K., Kanamitsu, M., Noone, D. & Oki, T. Historical isotope simulation using reanalysis atmospheric data. *J. Geophys. Res.: Atmos.* **113** (2008).

2 Yang, H., Johnson, K., Griffiths, M. & Yoshimura, K. Interannual controls on oxygen isotope variability in Asian monsoon precipitation and implications for paleoclimate reconstructions. *J. Geophys. Res.: Atmos.* **121**, 8410-8428 (2016).

3 Fohlmeister, J. A statistical approach to construct composite climate records of dated archives. *Quat. Geochronol.* **14**, 48-56 (2012).

4 Zhang, N. *et al.* Timing and duration of the East Asian summer monsoon maximum during the Holocene based on stalagmite data from North China. *The Hol.* **28**, 1631-1641 (2018).

5 Zhao, K. *et al.* Contribution of ENSO variability to the East Asian summer monsoon in the late Holocene. *Palaeogeo, Palaeoclim., Palaeoecol.* **449**, 510-519 (2016).

6 Hu, C. *et al.* Quantification of Holocene Asian monsoon rainfall from spatially separated cave records. *Earth Planet. Sci. Lett.* **266**, 221-232 (2008).

7 Dykoski, C. A. *et al.* A high-resolution, absolute-dated Holocene and deglacial Asian monsoon record from Dongge Cave, China. *Earth Planet. Sci. Lett.* **233**, 71-86 (2005).

8 Hillman, A. L., Abbott, M. B., Finkenbinder, M. S. & Yu, J. An 8,600 year lacustrine record of summer monsoon variability from Yunnan, China. *Quat. Sci. Rev.* **174**, 120-132 (2017).

9 Wohlfarth, B. *et al.* Holocene environmental changes in northeast Thailand as reconstructed from a tropical wetland. *Global Planet. Change* **92**, 148-161 (2012).

10 Bond, G. *et al.* Persistent solar influence on north Atlantic climate during the Holocene. *Science* **294**, 2130-2136 (2001).

11 Tierney, J. E. & Pausata, F. S. Rainfall regimes of the Green Sahara. *Sci. adv.* **3**, e1601503 (2017).

12 McGee, D., Winckler, G., Stuut, J. & Bradtmiller, L. The magnitude, timing and abruptness of changes in North African dust deposition over the last 20,000 yr. *Earth Planet. Sci. Lett.* **371**, 163-176 (2013).

13 Cheng, H. *et al.* Improvements in 230Th dating, 230Th and 234U half-life values, and U–Th isotopic measurements by multi-collector inductively coupled plasma mass spectrometry. *Earth Planet. Sci. Lett.* **371**, 82-91 (2013).

14 Zhang, H. *et al.* Hydroclimatic variations in southeastern China during the 4.2 ka event reflected by stalagmite records. *Clim. Past* **14**, 1805-1817 (2018).

15 Zhang, M. *et al.* A 6000-year high-resolution climatic record from a stalagmite in Xiangshui Cave, Guilin, China. *The Hol.* **14**, 697-702 (2004).

16 Tan, L. *et al.* Centennial-to decadal-scale monsoon precipitation variations in the upper Hanjiang River region, China over the past 6650 years. *Earth Planet. Sci. Lett.* **482**, 580-590 (2018).

17 Kubota, Y. *et al.* Variations of East Asian summer monsoon since the last deglaciation based on Mg/Ca and oxygen isotope of planktic foraminifera in the northern East China Sea. *Paleocean.* **25** (2010).

18 Zhang, E. *et al.* Millennial-scale hydroclimate variations in southwest China linked to tropical Indian Ocean since the Last Glacial Maximum. *Geology* **45**, 435-438 (2017).

19 Wu, L. *et al.* Mid-Holocene palaeoflood events recorded at the Zhongqiao Neolithic cultural site in the Jianghan Plain, middle Yangtze River Valley, China. *Quat. Sci. Rev.* **173**, 145-160 (2017).

20 Zhao, L. *et al.* Holocene vegetation dynamics in response to climate change and human activities derived from pollen and charcoal records from southeastern China. *Palaeogeo, Palaeoclim., Palaeoecol.* **485**, 644-660 (2017).

21 Zhou, W. *et al.* High-resolution evidence from southern China of an early Holocene optimum and a mid-Holocene dry event during the past 18,000 years. *Quat. Res.* **62**, 39-48 (2004).

22 Zhong, W. *et al.* Climatic changes since the last deglaciation inferred from a lacustrine sedimentary sequence in the eastern Nanling Mountains, south China. *J. Quat. Sci.* **25**, 975-984 (2010).

23 Ma, C. *et al.* High-resolution geochemistry records of climate changes since late-glacial from Dajiuhu peat in Shennongjia Mountains, Central China. *Chin. Sci. Bull.* **53**, 28-41 (2008).

24 Huang, X. *et al.* Response of carbon cycle to drier conditions in the mid-Holocene in central China. *Nat. comm.* **9**, 1369 (2018).

25 Zhong, W., Cao, J., Xue, J. & Ouyang, J. A 15,400-year record of climate variation from a subalpine lacustrine sedimentary sequence in the western Nanling Mountains in South China. *Quat. Res.* **84**, 246-254 (2015).

26 Yue, Y. *et al.* A continuous record of vegetation and climate change over the past 50,000 years in the Fujian Province of eastern subtropical China. *Palaeogeo, Palaeoclim., Palaeoecol.* **365**, 115-123 (2012).

27 Huang, C. C. *et al.* Extraordinary floods of 4100− 4000 a BP recorded at the Late Neolithic ruins in the Jinghe River gorges, Middle Reach of the Yellow River, China. *Palaeogeo, Palaeoclim., Palaeoecol.* **289**, 1-9 (2010).

28 Huang, C. C., Pang, J., Zha, X., Su, H. & Jia, Y. Extraordinary floods related to the climatic event at 4200 a BP on the Qishuihe River, middle reaches of the Yellow River, China. *Quat. Sci. Rev.* **30**, 460-468 (2011).

29 Tan, M. & Cai, B. Preliminary calibration of stalagmite oxygen isotopes from eastern monsoon China with Northern Hemisphere temperatures. *Pages News* **13**, 16-17 (2005).

30 Wang, Y. *et al.* The Holocene Asian monsoon: links to solar changes and North Atlantic climate. *Science* **308**, 854-857 (2005).

31 Jiang, X., He, Y., Shen, C.-C., Li, Z. & Lin, K. Replicated stalagmite-inferred centennial-to decadal-scale monsoon precipitation variability in southwest China since the mid Holocene. *The Hol.* **23**, 841-849 (2013).

32 Berkelhammer, M. *et al.* An abrupt shift in the Indian monsoon 4000 years ago. *Geophys. Monogr. Ser* **198**, 75-87 (2012).

33 Xiao, J. *et al.* The 4.2 ka event and its resulting cultural interruption in the Daihai Lake basin at the East Asian summer monsoon margin. *Quat. Int.* (2018).

34 Zhou, J., Wang, S. & Jing, L. Climatic and environmental changes from the sediment record of Erhai Lake over the past 10000 years. *J. Lake Sci.* **15**, 104-111 (2003).

35 Zhao, Y., Chen, F., Zhou, A., Yu, Z. & Zhang, K. Vegetation history, climate change and human activities over the last 6200 years on the Liupan Mountains in the southwestern Loess Plateau in central China. *Palaeogeo, Palaeoclim., Palaeoecol.* **293**, 197-205 (2010).

36 Chen, F. *et al.* East Asian summer monsoon precipitation variability since the last deglaciation. *Sci. rep.* **5**, 11186 (2015).

37 Xiao, J., Si, B., Zhai, D., Itoh, S. & Lomtatidze, Z. Hydrology of Dali lake in central-eastern Inner Mongolia and Holocene East Asian monsoon variability. *J. Paleolim.* **40**, 519-528 (2008).

38 Xiao, J. *et al.* The 4.2 ka BP event: multi-proxy records from a closed lake in the northern margin of the East Asian summer monsoon. *Clim. Past* **14**, 1417-1425 (2018).

39 Yao, F. *et al.* Holocene climate change in the western part of Taihu Lake region, East China. *Palaeogeo, Palaeoclim., Palaeoecol.* **485**, 963-973 (2017).

40 Yancheva, G. *et al.* Influence of the intertropical convergence zone on the East Asian monsoon. *Nature* **445**, 74 (2007).

41 Hong, Y. *et al.* Correlation between Indian Ocean summer monsoon and North Atlantic climate during the Holocene. *Earth Planet. Sci. Lett.* **211**, 371-380 (2003).
